# Histidine and other amino acids in blood and urine after administration of Bretschneider solution (HTK) for cardioplegic arrest in patients: effects on N-metabolism

**DOI:** 10.1007/s00726-016-2195-2

**Published:** 2016-02-27

**Authors:** Johanna K. Teloh, Daniel-Sebastian Dohle, Miriam Petersen, Rabea Verhaegh, Indra N. Waack, Friederike Roehrborn, Heinz Jakob, Herbert de Groot

**Affiliations:** Institute of Physiological Chemistry, University Hospital Essen, University of Duisburg-Essen, Hufelandstraße 55, 45122 Essen, Germany; Department of Thoracic and Cardiovascular Surgery, University Hospital Essen, Hufelandstraße 55, 45122 Essen, Germany; Ambulatory Healthcare Center MVZ Dr. Eberhard und Partner, Brauhausstraße 4, 44137 Dortmund, Germany

**Keywords:** Cardioplegia, Cardiopulmonary bypass, Heart–lung machine, Inzolen

## Abstract

Bretschneider (histidine-tryptophan-ketoglutarate, HTK) solution employed for induction of cardioplegic arrest possesses a high histidine concentration (198 mM). Due to the large volume administered, massive amounts of histidine are incorporated. The aim of the study was to evaluate alterations in amino acid and nitrogen metabolism originating from histidine degradation. Between 07/2014 and 10/2014, a total of 29 consecutive patients scheduled for elective isolated coronary artery bypass grafting with cardiopulmonary bypass (CPB) were enrolled in this prospective observational study. The patients received 1.6 L cardioplegic Bretschneider solution on average. Blood gas and urine samples obtained were analyzed for amino acid as well as urea and ammonium concentrations. After CPB initiation, plasma histidine concentration greatly increased to 21,000 µM to reach 8000 µM at the end. Within the operative period, plasma concentrations of aspartate, glutamate, asparagine, alanine, and glutamine increased variable in magnitude. During the same time, urinary analysis revealed histidine excretion of 19,500 µmol in total and marked elevations in glutamate and glutamine excretion. The absolute amounts of urea and ammonium excreted additionally were 3 mmol and 8 mmol, respectively. Already during CPB, distinct amounts of the histidine administered are metabolized, mainly to other amino acids, but only small amounts to urea and ammonia. Thus, the impact of the histidine incorporated on acid–base status in the intraoperative phase is minor. On the other hand, intraoperative provision of several amino acids arising from histidine metabolism might mitigate postaggression syndrome.

## Introduction

Bretschneider (histidine-tryptophan-ketoglutarate, HTK) solution is routinely administered for cardioplegic arrest in many countries (Careaga et al. 2001). In clinical studies as well as in experimental models the Bretschneider solution has been shown to achieve a better myocardial protection during ischemia as compared to pure electrolyte-containing crystalloid cardioplegic solutions without histidine or to blood cardioplegia (Beyersdorf et al. 1990; Careaga et al. 2001; Kober et al. 1998; Korun et al. 2013; Sakata et al. 1998; Scrascia et al. 2011). The effectiveness of the solution becomes apparent in an increased cardiac output, fewer arrhythmias, more frequent spontaneous defibrillation as well as decreasing length of stay in the intensive care unit (Careaga et al. 2001; Sakata et al. 1998). The included histidine (198 mM) is associated with enhanced efficiency of anaerobic glycolysis due to a high buffer capacity, whereas α-ketoglutarate (1 mM), an intermediary of the Krebs cycle, is assumed to serve energy production. Tryptophan (2 mM) and mannitol (30 mM) are proposed to function as a stabilizer of cell membranes and to reduce cellular edema formation, respectively (Careaga et al. 2001). Usually, if Bretschneider solution is employed, almost all of the administered volume enters the systemic circulation. Thus, due to the large volume of the Bretschneider solution applied for induction of cardioplegic arrest, there is a massive incorporation of histidine.

To date, only very few data exist on plasma amino acid concentrations originating from histidine metabolism related to Bretschneider cardioplegia (Doetsch et al. 1987; Schayani-Mühlschlegel 1990). Therefore, we will here analyze concentrations of especially histidine but also further amino acids as well as urea and ammonia in plasma derived from intraoperatively taken blood gas samples as well as urine samples obtained at the beginning and the end of the operation, thus trying to establish an overall balance of amino acid and nitrogen metabolism under these conditions.

## Materials and methods

### Study design and patient population

Between 07/2014 and 10/2014, a total of 29 consecutive patients scheduled for elective isolated coronary artery bypass grafting (CABG) with cardiopulmonary bypass (CPB) were enrolled in the prospective observational designed study at the Department of Thoracic and Cardiovascular Surgery, University Hospital Essen. The study was approved by the Medical Ethics Committee of the University Hospital Essen and confirms to the principles of the Declaration of Helsinki. All individuals gave written informed consent. In short, myocardial protection was achieved using antegrade cold crystalloid Bretschneider cardioplegia (Custodiol, Dr. Franz Koehler Chemie, Bensheim, Germany), employing 1.6 ± 0.2 L on average supplemented by topical cooling, and single aortic cross-clamping for all distal anastomoses. After weaning from the heart–lung machine, patients received 56 mL Inzolen (Dr. Franz Koehler Chemie, Bensheim, Germany) on average. For further details see (Teloh et al. 2015).

### Patient Characteristics

Of all patients, 76 % were male gender. The median values for age, height, weight, cardiopulmonary bypass time, and cross-clamp-time were 71 years, 173 cm, 84 kg, 86 min and 53 min, respectively. On average, patients received three grafts each.

### Data collection

Blood gas samples were routinely taken during operative procedures (initially, after beginning of CPB, before cessation of CPB, after cessation of CPB, before the end of operative procedures), and centrifuged at 3000*g* for 10 min at room temperature. Subsequently, the plasma was taken off and stored at −80 °C until analysis.

Immediately after catheterization of the patient’s urinary bladder, a urine sample was obtained in order to represent baseline conditions. At the end of the operative procedures, a second sample was gathered from the volume that had been collected during the operation.

### Measurements

For ammonium quantification in urine, capillary electrophoresis (P/ACE MDQ, Beckmann Coulter, Krefeld, Germany) was used. For this purpose, a fused silica capillary was employed with an effective length of 50 cm, an I.D. of 75 µm and an O.D. of 375 µm. Samples of initial urine were diluted with ultrapure water 1:50. Analysis was performed using a cation analysis kit (ABSciex, Fullerton, USA) and pressure injection. The subsequent separation proceeded using a voltage of 30 kV and normal polarity of the capillary. Indirect detection was performed employing a photo diode array at a wavelength of 200 nm. Due to the low sensitivity of the employed capillary electrophoresis, ammonium quantification in plasma was performed with an enzymatic method in the central laboratory of the University Hospital Essen.

Urine and plasma were analyzed for urea. Plasma samples were diluted 1:4, whereas urine was diluted 1:10 with 0.9 % NaCl. Urea was determined with the help of a fully automated clinical chemistry analyzer (Respons 920, DiaSys Diagnostics, Holzheim, Germany) using a commercially available reagent (DiaSys Diagnostics, Holzheim, Germany) for the enzymatic reactions of urease and glutamate dehydrogenase and subsequent detection of NADH decrease at 340 nm.

Urine and plasma samples were also analyzed for amino acids. For deproteinization, 300 µL sample were added to 75 µL sulfosalicylic acid (10 % in water) and thoroughly mixed. This mixture was centrifuged at 12,000 U/min for 5 min. Subsequently, 200 µL of the supernatant were diluted with reagent buffer at the ratio of 1:1. Of this formulation, 50 µL were injected into the liquid chromatograph (biochrom 30+, biochrom, Cambridge, UK). With the help of the employed cation exchanger, separation of amino acids took place at a rate of 0.25 mL/min due to varying pH and molarity of used running buffers containing citrate. Following this, the amino group reacted with ninhydrin, forming a colored complex which was detected at 570 nm (primary amino acids) and 440 nm (secondary amino acids), respectively. Urine as well as plasma samples were treated in the same way. Due to technical limitations in association with the high histidine concentration in plasma subsequent to Bretschneider application, tryptophan could not be exactly quantified.

### Statistical analysis

All data are expressed as mean values ± standard deviation (SD) unless otherwise stated. Comparisons among different time points were performed using one-way independent analysis of variance (ANOVA) followed by the Fisher (LSD) post hoc analysis. A *P* value <0.05 was considered significant.

## Results

**Fig. 1:**
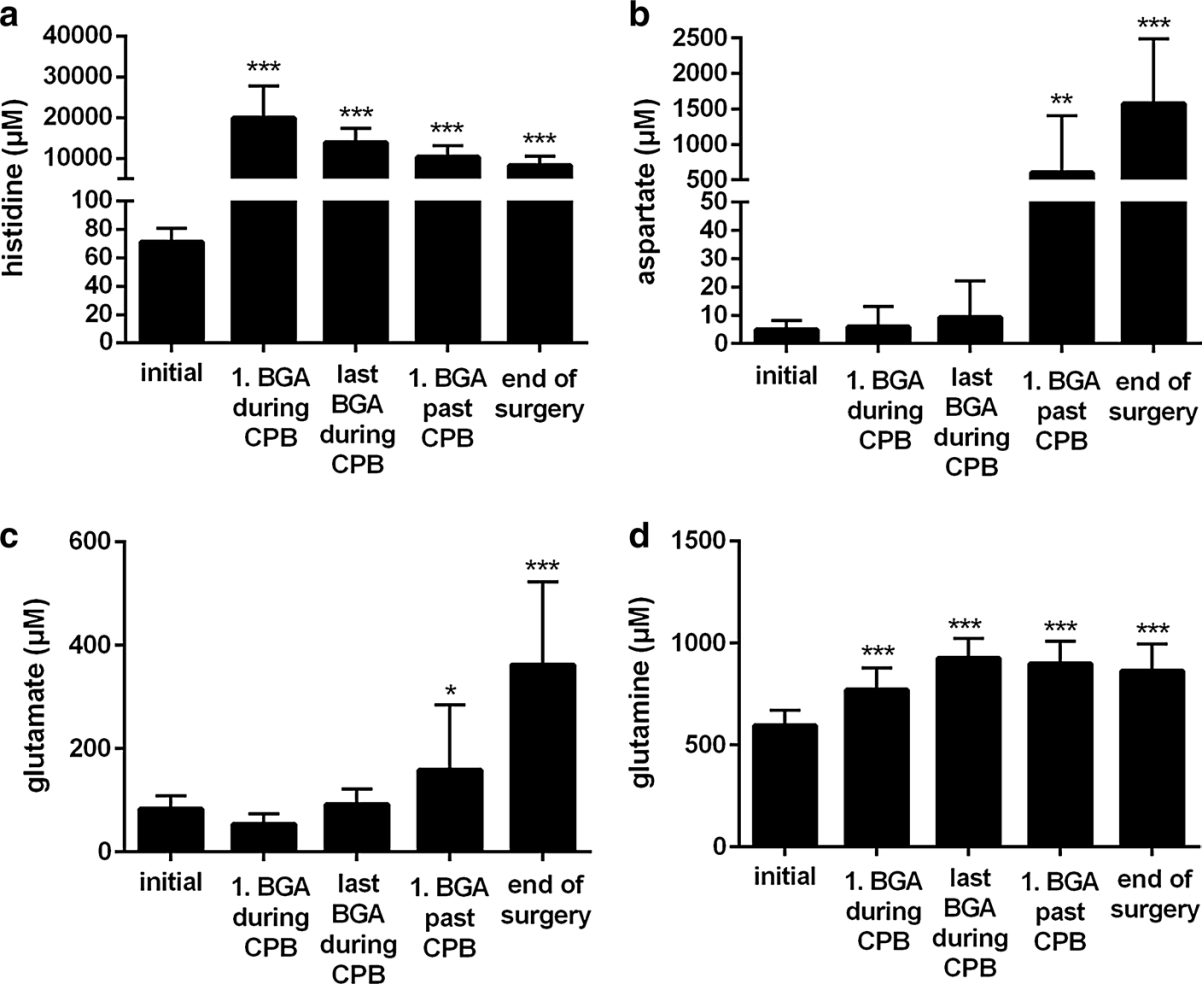
Intraoperative plasma concentrations of **a** histidine, **b** aspartate, **c** glutamate, **d** glutamine. Patients received 1.6 L cardioplegic solution at the onset of cardiopulmonary bypass, which contained 198 mM histidine for induction of cardioplegia. Values are shown as mean ± SD. *Asterisk* <0.05 compared with the initial value. *Double asterisk* <0.01 compared with the initial value. *Triple asterisk* <0.001 compared with the initial value

**Fig. 2:**
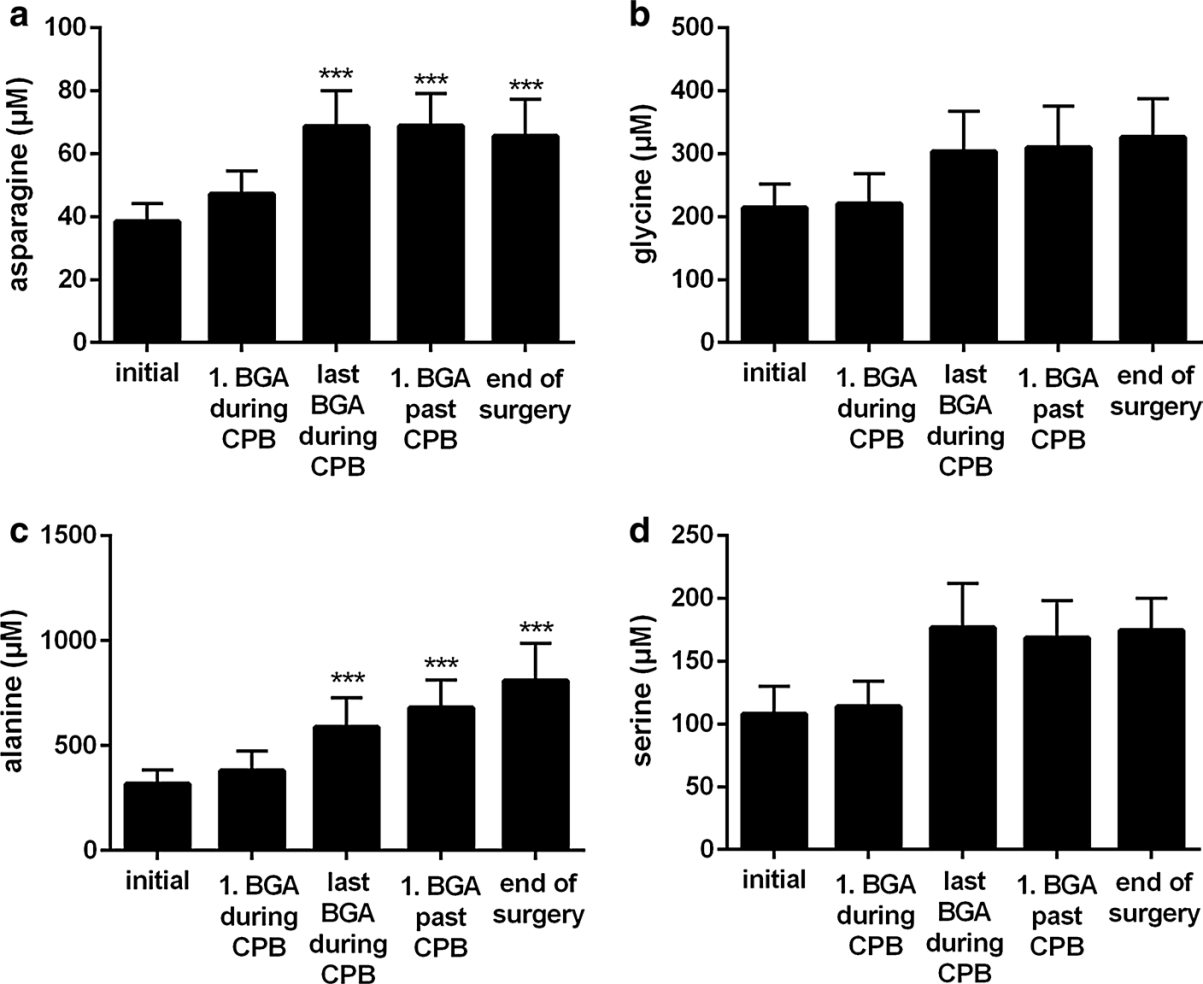
Intraoperative plasma concentrations of **a** asparagine, **b** glycine, **c** alanine, **d** serine. Patients received 1.6 L cardioplegic solution at the onset of cardiopulmonary bypass, which contained 198 mM histidine for induction of cardioplegia. Values are shown as mean ± SD. *Triple asterisk* <0.001 compared with the initial value

After start of CPB with concomitant induction of cardioplegic arrest, plasma histidine concentration sharply increased from an initial value of 71 to 21000 µM (Fig. 1a). Subsequently, during the course of the operation, it steadily decreased, reaching a concentration of 8000 µM at the end. The aspartate’s concentration in plasma rose from 5 µM before to 1600 µM at the end of operation (Fig. 1b). Within the same interval, plasma glutamate concentration increased from 23 to 360 µM (Fig. 1c). Plasma concentrations of glutamine, asparagine, glycine, alanine and serine rose modestly from initial values of 596, 38, 214, 318, and 108 µM, respectively, to 862, 65, 325, 807, and 174 µM, respectively, at the end of the operation (Figs. 1d, 2a–d). Plasma concentrations of arginine, leucine, lysine, methionine, ornithine, phenylalanine, proline, tyrosine, threonine as well as valine varied little, and stayed within the respective reference ranges (Table 1).

**Table 1:** Intraoperative plasma concentrations of different amino acids. From (Duran 2008)

Amino acid	Initial	1. BGA during CPB	Last BGA during CPB	1. BGA past CPB	End of operation	Reference range
Arginine (µM)	71 ± 18	70 ± 8	90 ± 20	81 ± 21	71 ± 19	15–190
Leucine (µM)	135 ± 27	174 ± 30	196 ± 36	173 ± 34	159 ± 35	70–200
Lysine (µM)	179 ± 21	192 ± 28	222 ± 39	207 ± 37	194 ± 31	115–300
Methionine (µM)	22 ± 4	24 ± 5	27 ± 6	24 ± 5	25 ± 6	10–40
Ornithin (µM)	54 ± 15	49 ± 16	57 ± 15	55 ± 17	52 ± 22	50–200
Phenylalanine (µM)	55 ± 7	53 ± 9	44 ± 13	42 ± 12	37 ± 10	35–85
Proline (µM)	149 ± 28	151 ± 32	185 ± 22	182 ± 30	201 ± 39	97–330
Tyrosine (µM)	56 ± 11	58 ± 11	54 ± 12	51 ± 11	47 ± 9	35–115
Threonine (µM)	117 ± 31	131 ± 31	176 ± 41	173 ± 36	170 ± 36	60–225
Valine (µM)	237 ± 28	254 ± 37	271 ± 39	263 ± 43	258 ± 38	120–340

Values are given as mean value ± SD

**Fig. 3:**
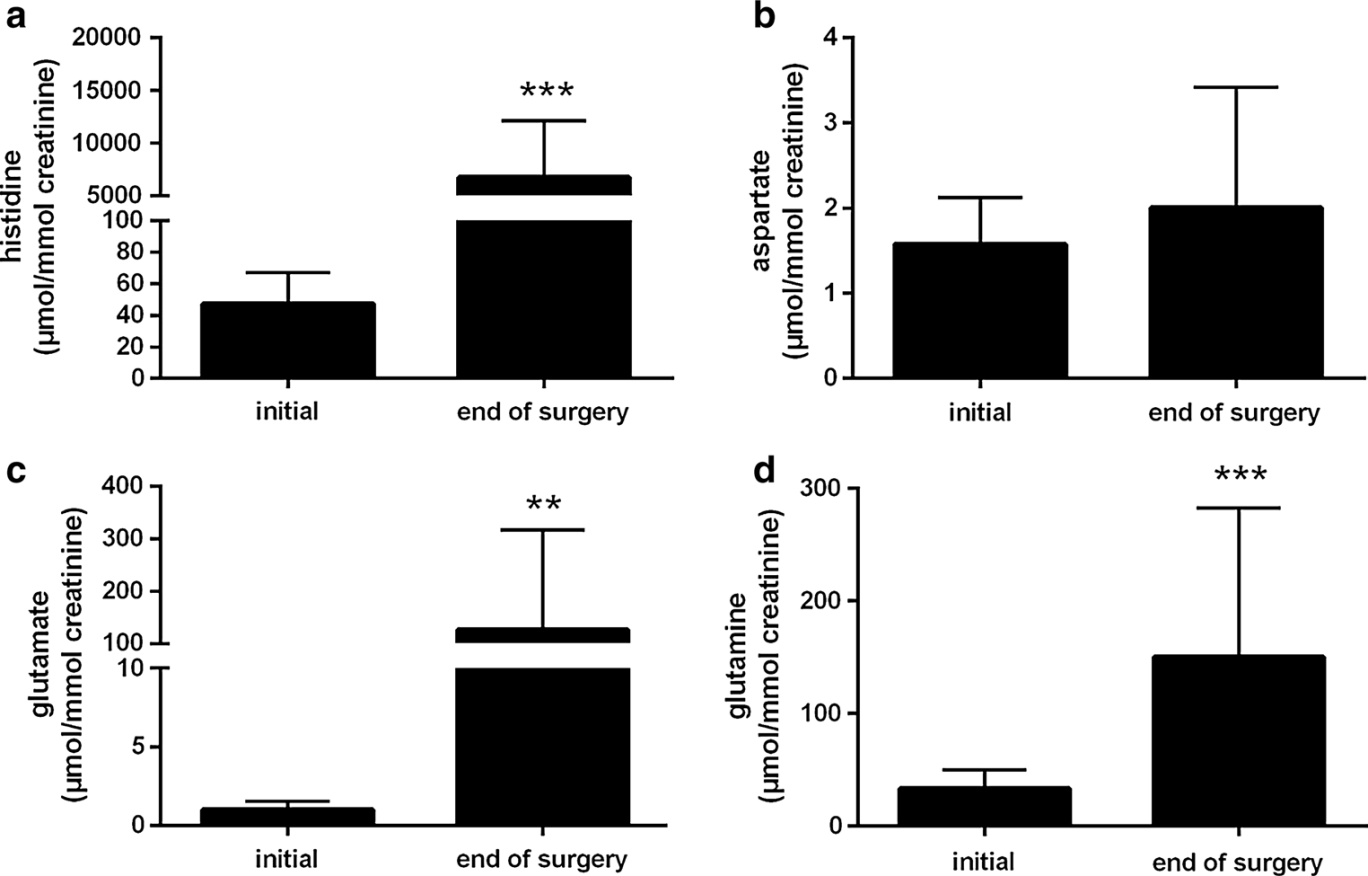
Excreted amounts of **a** histidine, **b** aspartate, **c** glutamate, **d** glutamine during the intraoperative phase. Values are shown as mean ± SD. *Double asterisk* <0.01 compared with the initial value. *Triple asterisk* <0.001 compared with the initial value

**Fig. 4:**
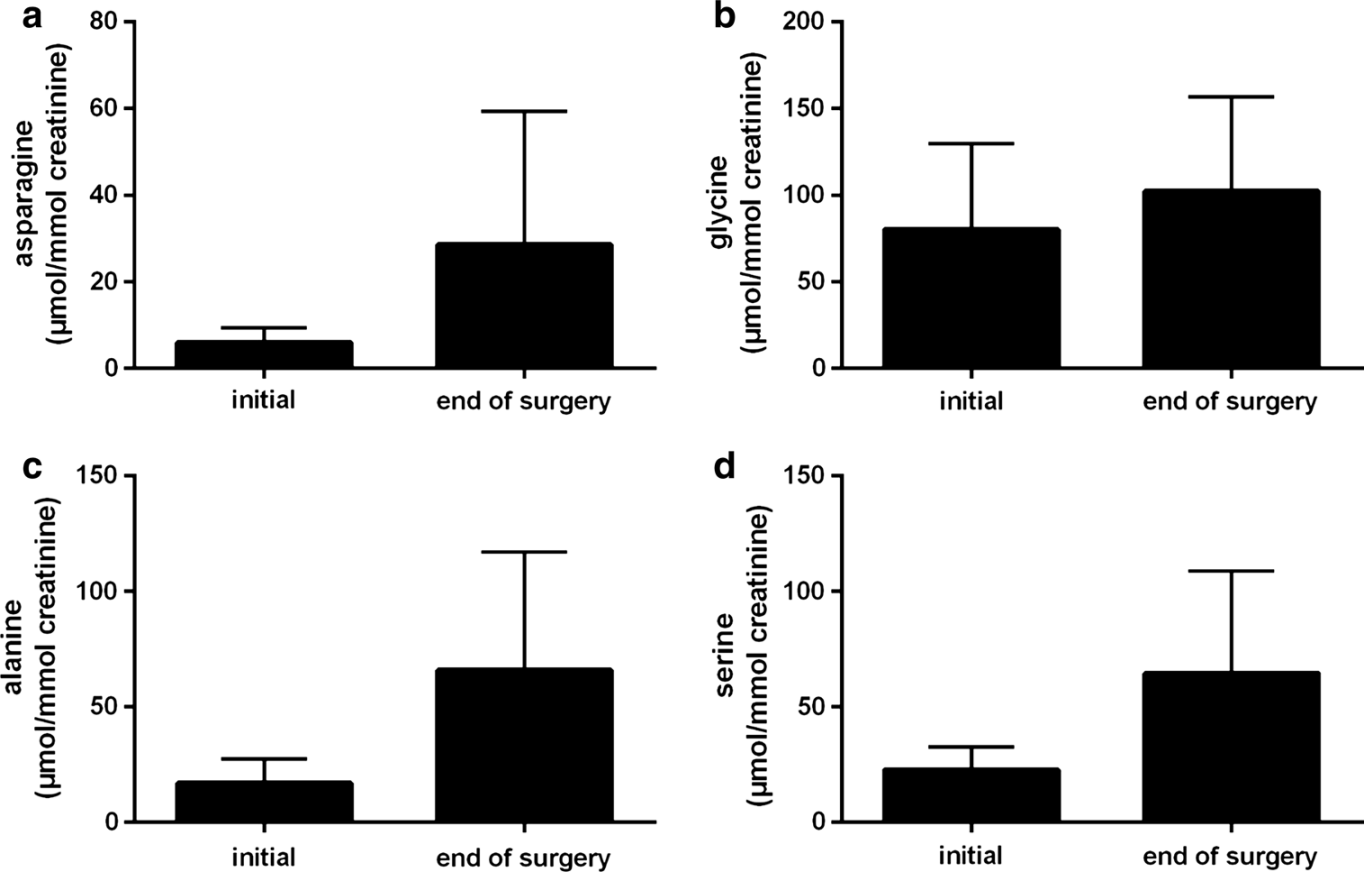
Excreted amounts of **a** asparagine, **b** glycine, **c** alanine, **d** serine during the intraoperative phase. Values are shown as mean ± SD

Histidine excretion largely increased from 47 µmol/mmol creatinine at basal conditions to 6760 µmol/mmol creatinine at the end of operation (Fig. 3a). Taking the intraoperatively excreted urine volume into account, it amounted to 19.5 mmol, i.e. just under 7 % of the incorporated amount of histidine (300 mmol). In the same interval, glutamate excretion rose from 1 µmol/mmol creatinine to 126 µmol/mmol creatinine and glutamine excretion from 33 µmol/mmol creatinine to 150 µmol/mmol creatinine (Fig. 3c, d). Excretion of aspartate, asparagine, glycine, alanine and serine slightly increased as well but stayed within the reference range (Figs. 3b, 4a–d). Amounts of carnosine, 1-methylhistidine and 3-methylhistidine in urine were subjected to only minimal changes (data not shown).

**Fig. 5:**
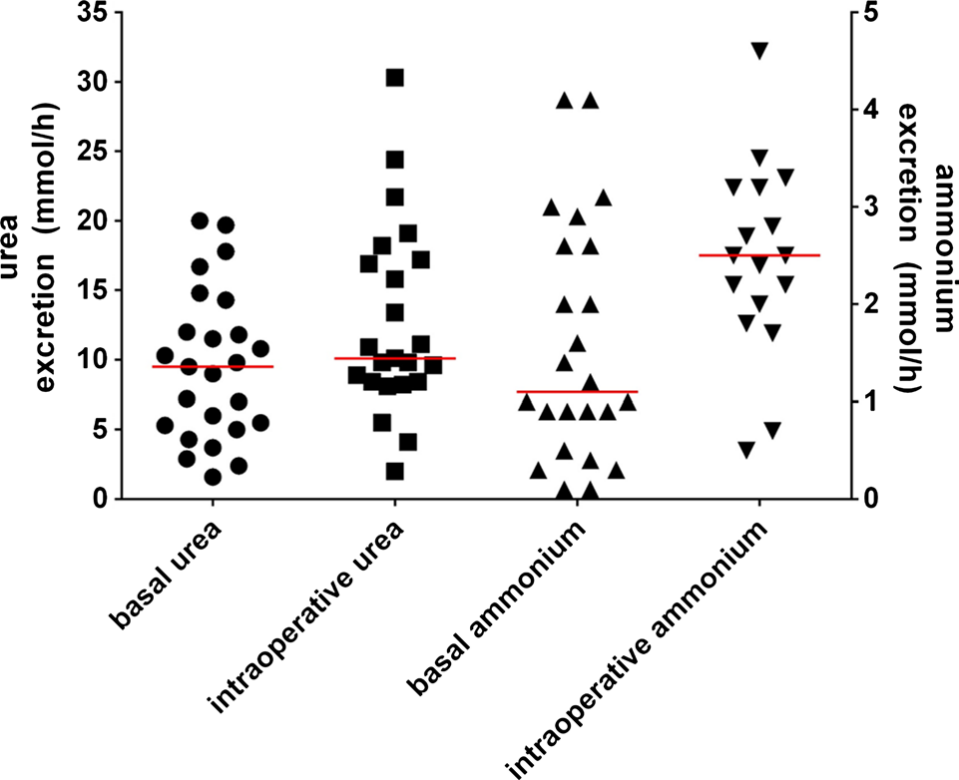
Urea as well as ammonium excretion per hour from every patient at basal conditions and in the intraoperative interval, respectively. *Lines* represent median

Urea and ammonium excretion per hour increased during the time of operative procedures from 9.5 and 1.1 mmol/h, respectively, at basal conditions to 10.1 and 2.6 mmol/h, respectively (Fig. 5). This increase in excretion amounts to 3 mmol urea and 8 mmol ammonium (both median) in the intraoperative interval compared to basal excretion. The magnitude of increase in urea excretion differed among patients, thus leading to fluctuating values in the range of 2–30 mmol/h. In plasma, over the course of the operation, the median of urea concentration was about 30 mg/dL (5 mM). Plasma ammonium concentration represented about 90 µg/dL (50 µM).

## Discussion

In the human organism, histidine is degraded by two major pathways (Bender 2012a; Doetsch et al. 1987). First, it can be deaminated to glutamate via urocanic acid, which is supposed to be the dominant one. Second, it can be decarboxylated to finally yield aspartate. As soon as these two pathways that ensure a specific degradation are exhausted due to a high histidine concentration in plasma, other pathways are activated in addition, yielding those amino acids with short unbranched side chains while maintaining the histidine’s α-amino-carboxylic acid group (Doetsch et al. 1987). That way, glycine, alanine or serine may be obtained, depending on the length of the hydrocarbon chain, and a possible hydroxylation.

**Fig. 6:**
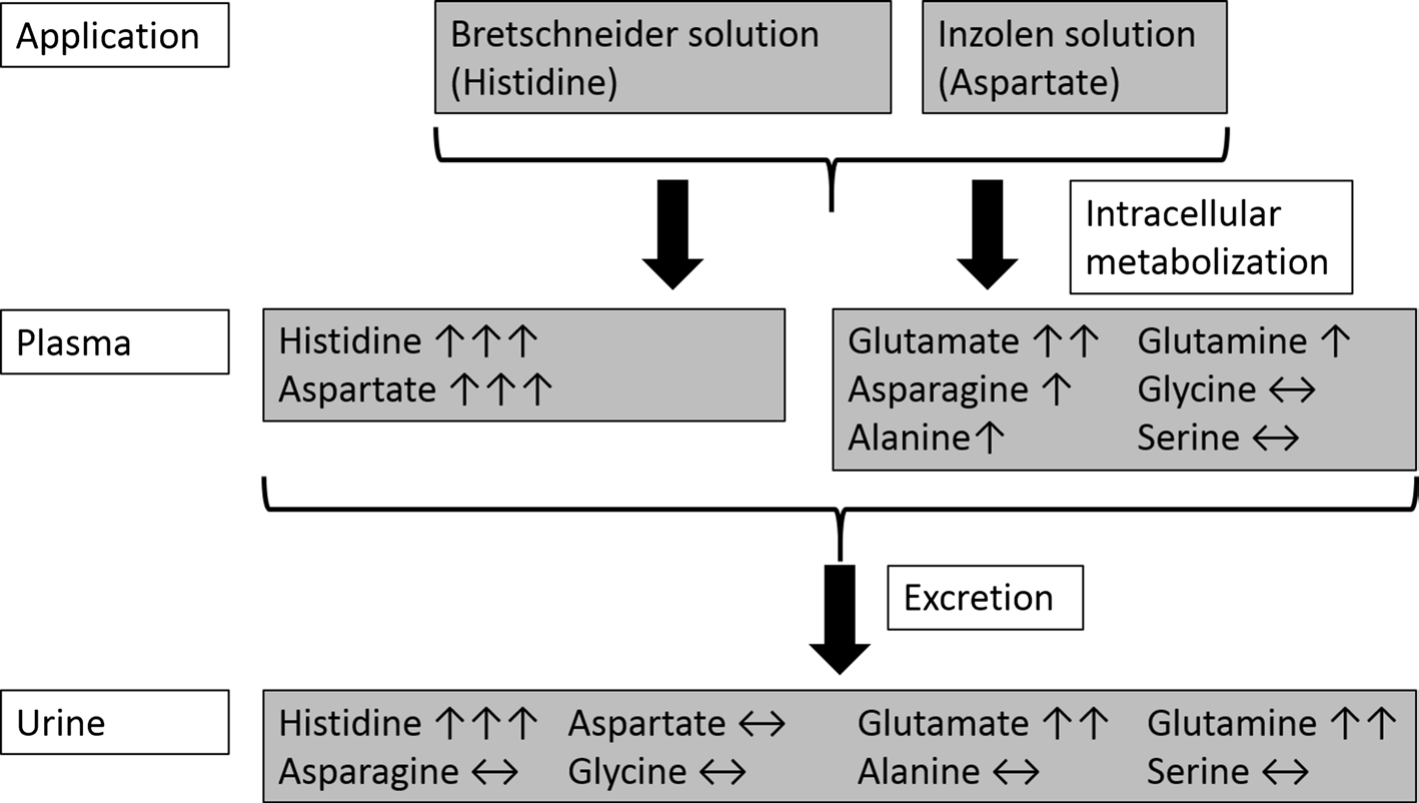
Survey of application, metabolization and renal excretion of several amino acids during cardiopulmoanry bypass (CPB) in coronary artery bypass grafting patients. Patients received Bretschneider solution (main component histidine) for induction of cardioplegic arrest and Inzolen solution (main component potassium aspartate) after weaning from CPB to correct a shortage of potassium. In the course of intracellular metabolization, several amino acids emerge that show up in plasma, some of them are renally excreted. ↑↑↑, strong concentration increase; ↑↑, indicates moderate concentration increase; ↑, indicates slight concentration increase; ↔, indicates no change or changes staying within the reference range

Patients received 1.6 L Bretschneider solution on average for induction of cardioplegic arrest. Based on the applied volume and its histidine concentration of 198 mM, a total of about 300 mmol histidine had been incorporated (Fig. 6). Assuming an estimated blood volume of approximately 5.2 L [calculation on the basis of the formula of Nadler (Nadler et al. 1962)], a plasma concentration of about 60 mM would have to be expected. However, only about a third of this calculated concentration, i.e. 20 mM, was detected in plasma in accordance with two former studies (Doetsch et al. 1987; Schayani-Mühlschlegel 1990). This strongly suggests the participation of the interstitial space for distribution. Actually, inclusion of the entire extracellular volume for calculation [intravascular plus interstitial, approximately 2.9-fold the blood volume (Grocott et al. 2005)] would result in a histidine concentration of approximately 20 mM. Thus, obviously, soon after incorporation (first sample during CPB), an equilibrium between blood and the interstitial space had already been achieved.

The physiologic histidine concentration in plasma accounts for approximately 100 µM (Table 2). Under these conditions, 5 % of the filtered histidine are renally excreted due to major reabsorption in the proximal tubule (Lingard et al. 1973; Silbernagl and Volkl 1977), i.e. about 100 µmol/mmol creatinine (Table 2). In the present study, attributable to the highly elevated plasma histidine level (20 mM), the excreted amount accounted for 6800 µmol/mmol creatinine during the intraoperative interval. This value (equivalent to an absolute amount of 19.5 mmol histidine) corresponds to 7 % of the incorporated histidine (300 mmol). For comparison, Doetsch et al. reported an amount of histidine excreted within the first 72 postoperative hours representing 20 % of the administered dose (Doetsch et al. 1987). During the intraoperative phase, plasma histidine concentration decreased from 20 mM to 8 mM. Renal excretion contributes only to a minor extent to this decrease (19.5 mmol/extracellular space of 15.1 L = 1.3 mM). Thus, uptake into cells and metabolic degradation (see below) should mainly be responsible for the rapid decline.

**Table 2:** Reference ranges of different amino acids, urea and ammonia in plasma as well as amino acids in urine

	Reference range
Plasma	
Histidine	70–125 µM
Aspartate	0–25 µM
Glutamate	10–130 µM
Glutamine	200–760 µM
Asparagine	35–75 µM
Glycine	150–490 µM
Alanine	175–580 µM
Serine	60–180 µM
Urea	17–43 mg/dL
Ammonia	27–90 µg/dL
Urine (µmol/mmol creatinine)	
Histidine	52–162
Aspartate	5–27
Glutamate	5–37
Glutamine	22–58
Asparagine	11–53
Glycine	83–475
Alanine	27–76
Serine	27–76

From Duran (2008), Thomas (1998) and Waters et al. (1967)

In accordance with the histidine’s main degradation pathways (see above), plasma concentrations of both aspartate and glutamate increased during the operation (Fig. 1b, c). Interestingly, however, the increase in plasma aspartate concentration was clearly more pronounced than the increase in plasma glutamate concentration (about 1600 µM at the end of the operation, 63-times above the upper limit of the reference range vs. 360 µM which is 2.8-times above the upper limit of the reference range, Table 2), although the degradation pathway yielding glutamate is supposed to constitute the major route (Ghadimi 1974; Mehler and Tabor 1953). A plausible explanation for this unexpected behavior in plasma aspartate concentration is the application of Inzolen (Fig. 6). In conjunction with cardiac surgery, Inzolen solution, consisting mainly of racemic potassium aspartate and further trace elements, is usually applied to correct a shortage of potassium. In the present study, due to the solution’s composition, patients received approximately 24 mmol aspartate after weaning from CPB this way. Despite the aspartate’s highly elevated plasma level, urine analysis revealed only minor excretion (Fig. 3b), but enhanced excretion of glutamate (126 µmol/mmol creatinine at the end of operative procedures, 3-times the upper limit of the reference range, Fig. 3c; Table 2). Because aspartate and glutamate possess the same net charge at physiological pH, they are reabsorbed by the same carrier in the proximal tubule [in the case of aspartate independent of the isomer (Silbernagl 1983; Silbernagl and Volkl 1983)]. Obviously, reabsorption of aspartate is preferred which is in line with the higher affinity of the carrier for aspartate (K_M_ 0.10 mM for aspartate vs. 0.17 mM to 0.50 mM for glutamate, both determined in the rat) (Silbernagl 1981, 1983).

Those amino acids having been formed by side chain conversion while maintaining the histidine’s original α-amino-carboxylic acid group, i.e. glycine, alanine and serine, also increased in plasma in the course of the operation, although variable in magnitude, with glycine and serine staying within the reference interval (Fig. 2b–d; Table 2). The increase of alanine (300 µM to 800 µM at the end of the operation, 1.4-times above the upper limit of the reference range, Table 2) was plainest among those three amino acids, perhaps either due to the transamination reaction with glutamate or with histidine itself. The latter reaction occurs rarely under physiologic conditions but becomes more important in diseases associated with histidinemia (Bender 2012a), characterized by histidine plasma levels up to 1.8 mM (Ghadimi 1974; Virmani and Widhalm 1993). In accordance with the elevated concentrations in plasma, urinary excretion of glycine, alanine and serine increased slightly during the intraoperative interval, but stayed within the reference interval (Fig. 4b–d; Table 2). Glutamine as well as asparagine possess the capability to accommodate an additional amino group, therefore representing the possibility to store further nitrogen. However, the plasma concentrations of glutamine as well as asparagine increased only moderately during the operation (Figs. 1d, 2a). For glutamine, this probably results from continuous catabolism for the purpose of ammonium synthesis and an increased excretion (150 µmol/mmol creatinine, 2.6-times the upper limit of the reference range; Fig. 3d; Table 2). Increased glutamine excretion might arise from end product inhibition of glutamine and glutamate catabolism by alpha-ketoglutarate (Yao et al. 2012). Carnosine (β-alanyl-histidine, <5 µM), 1-methylhistidine as well as 3-methylhistidine (both <3 µM) as further degradation products of histidine (Bender 2012a) stayed below the limit of quantification in plasma and were comparable to baseline conditions at the end of operative procedures in urine (data not shown). Thus, these pathways of histidine metabolism remained unused.

Alterations in glutamate, glutamine, alanine, asparagine and aspartate may arise from histidine metabolism. In addition, aspartate is applicated with the Inzolen solution. All are, directly or indirectly, glucoplastic amino acids (Bender 2012a, b). Therefore, they can be used for gluconeogenesis that is *per se* energy consuming. Energy consumption of asparagine, aspartate, glutamine and glutamate is smaller during this process compared to that of alanine, since pyruvate deriving from alanine degradation must first be carboxylated to oxaloacetate, which costs additional two molecules ATP per mol glucose. In contrast, metabolism of the aforementioned amino acids directly yields oxaloacetate or alpha-ketoglutarate that is converted to oxaloacetate via the citric acid cycle. Alpha-ketoglutarate deficiency in cardiac tissue occurs rapidly during ischemia (Peuhkurinen et al. 1983). Provision of alpha-ketoglutarate (as an additive in blood cardioplegia) has been shown to attenuate myocardial ischemic injury in patients undergoing coronary revascularization (Kjellman et al. 1995). Hence, exogenous supply might preserve myocardial oxidative capacity. In addition, it may help to minimize postoperative muscle catabolism (see below) (Wernerman et al. 1990). Thus, the addition of 1 mM alpha-ketoglutarate to the cardioplegic solution seems beneficial, despite the possible conversion of amino acids arising from histidine metabolism into alpha-ketoglutarate in the later course. The cardioplegic solution also contains 2 mM tryptophan. In the course of tryptophan metabolism, the antioxidant melatonin might be formed but also nicotinamide adenine dinucleotide might even increase which has also been reported to act as an antioxidant (Kirsch and de Groot 2001). Due to the melatonin’s inherent antioxidant function (Tan et al. 2002) but also the induction of antioxidant enzymes, it can exert cardio-protective effects against amongst others ischemia/reperfusion injury (Giacomo and Antonio 2007; Reiter and Tan 2003). Since during temporary cardioplegia the myocardium becomes ischemic very easily, this might have an appreciable contribution to myocardial protection during this phase as well.

The entire amount of histidine having been metabolized during the intraoperative period should be reflected by the differences in amino acid as well as urea and ammonium concentrations/amounts before (baseline values) and at the end of the operation in plasma as well as in urine, averaging 30 mmol in total. In this regard, the amount of further amino acids in urine except for histidine is negligible in terms of quantity. Of these 30 mmol, two-thirds account for the rise in several amino acids in plasma (19 mmol altogether) and one-third for elevation of ammonium (8 mmol) plus urea (3 mmol) excretion. Plasma urea and ammonia concentrations were in accordance with physiological values in the literature (Table 2), as expected for substances that are obligatory for excretion by urine. Hence, metabolism in this intraoperative phase is small, but principally, it is supposed to continue in the postoperative phase. In the special case of aspartate, this amino acid was left out of consideration. Due to Inzolen application beginning after weaning from CPB, the amount of aspartate rose continuously towards the end of the intraoperative period. For this reason, a considerable share originating from histidine metabolization is unlikely.

Apart from the α-amino group, every histidine possesses two additional nitrogen atoms located in the imidazole ring. In the course of metabolism, this nitrogen should be excreted either as ammonium ions or as urea in the long term. Nitrogen excretion *per se* is intimately linked to systemic acid–base status, since generation of urea is bicarbonate consuming (Han 2011; Meijer 1995; Pitts 1964), whereas ammonium is mainly obtained by deamination reactions from glutamine, glutamate or histidine (Han 2011; Pitts 1964; Weiner et al. 2015). As it is common for amino acid metabolism, the histidine’s α-amino group is converted to urea together with bicarbonate derived from the degradation of the remaining α-keto acid, thus being neutral as regards systemic acid–base homeostasis. The excretion of a surplus of 3 mmol urea (see above) should result in an additional base deficit of only −1 mEq/L. Thus, in relation to acute metabolic acidosis originating from massive dilution of endogenous bicarbonate based on administration of both the priming and the cardioplegic solution (Teloh et al. 2015), the impact of nitrogen metabolism on acid base status in the intraoperative phase is minor. Due to persistent metabolism in the postoperative phase, however, the acidifying effect caused by enhanced urea excretion might get more pronounced over the course of time. In contrast, metabolization of infused aspartate (24 mmol) as part of the Inzolen solution should have an alkalizing effect in general based on its additional carboxyl group.

Subsequent to severe trauma or operations, amongst others, a stress-induced increase in sympathetic nervous activity is observed, resulting in a hypermetabolic state, the so-called postaggression syndrome (Sachs et al. 1988). One of its characteristics is an increased gluconeogenesis from glucoplastic amino acids originating from enhanced protein degradation in skeletal muscle. Therefore, in such a condition, nitrogen balance is principally negative. Some evidence exist, that postaggression syndrome might be mitigated by amino acid administration. A benefit resulting from thereof is supported by a study of Umenai et al. that was able to show positive effects of perioperative amino acid infusion in patients undergoing off-pump CABG, resulting in a significantly shorter duration of postoperative mechanical ventilation as well as intensive care unit stay (Umenai et al. 2006). Therefore, intraoperative provision of several amino acids arising from histidine metabolism (19 mmol in the present study) might achieve a similar result, especially since many of them represent glucoplastic amino acids (see above). Endogenous protein sources like skeletal muscle might be spared from degradation this way. Because the provision of several amino acids takes place before the onset of postaggression syndrome, this application’s effect might be particularly beneficial.

In conclusion, in patients undergoing CABG, receiving approximately 1.6 L Bretschneider solution, a very substantial elevation of plasma histidine concentration was observed a few minutes after onset of CPB. Of the incorporated amount of 300 mmol histidine approximately 7 % were excreted without prior metabolization. In addition, about 10 % were metabolized, mostly being converted into other amino acids. Therefore, the influence on acid–base homeostasis originating from nitrogen metabolism is minor. Moreover, altered amino acid levels in plasma may have beneficial effects on postaggression syndrome in the postoperative phase.
